# Quality in peer review

**DOI:** 10.1038/s42003-019-0603-3

**Published:** 2019-09-20

## Abstract

The theme of this year’s Peer Review Week, Quality in Peer Review, reflects both the necessity of peer review and the growing uncertainty about its role in scholarly publishing. We support peer review that aims to improve manuscripts through critical evaluation before publication.

Peer review has been the subject of much discussion recently, particularly given that many researchers now choose to disseminate their work via online preprint servers (such as bioRxiv) prior to, or even instead of, traditional publication in a journal. Some have asked whether it’s necessary to have a manuscript evaluated by 2–3 experts before it can even see the light of day. Wouldn’t it be better to get it out quickly, and let the scientific community judge it for themselves?

There is merit to this idea. After all, traditional peer review is often slow and the quality of the reviews is unpredictable. There is no arguing the fact that quite a few papers are published with glaring errors and major flaws that were not caught by the reviewers or editors. But in our experience as editors, many more errors are identified during the prepublication peer review process and this leads to more robust papers making their way to journal pages. Of course, the evaluation of research by one’s peers doesn’t end at publication. Our position is that the less formal post-publication evaluation process cannot substitute for rigorous prepublication peer review.

When done well, peer review moves science forward. Independent journal editors help avoid potential conflicts of interest between reviewers and author. Unbiased reviewers provide useful critical feedback that authors can use to improve their work before sharing it with a broader audience. The purpose of peer review shouldn’t be to weed out all errors—this is close to impossible. Rather, it should help authors see their manuscript through the eyes of other experts while giving editors a clearer view as to whether the paper meets their journal’s criteria. At *Communications Biology*, we try to draw attention to the importance of quality peer review by highlighting some of our most constructive, thorough, and helpful referees on our Reviewer of the Month page.

When done well, peer review moves science forward.

But how do we ensure the quality of peer review? We have written before about the need for reviews to be fair and unbiased. Our Referees page also includes detailed guidelines for reviewers and links to reviewer training modules. But at the end of the day, it’s up to the reviewers themselves to approach the task with an open mind and a desire to help their colleagues produce the best possible science. It’s also up to the editors to request clarifications, provide clear guidance to authors, and (in rare cases) to redact improper comments. Editors can also help limit excessive cycles of review and revision.

The bottom line is that peer review works—but there is always room for improvement. This week, in honor of Peer Review Week, we are asking you to tell us: what does quality in peer review mean to you? We have published blog posts on various Nature Research community sites (communities.nature.com) featuring conversations with our Editorial Board members, who are authors, reviewers, and readers, in addition to their role as editors. To gain feedback from a wider community, we hosted a live Twitter chat this Thursday, September 19th, together with our sister journals *Communications Chemistry* and *Communications Physics*. Follow the hashtag #CommsChatPRW to read the comments and add your own. Finally, as always, we encourage you to comment on this editorial below to tell us your views on quality peer review.

